# New Insights into the Role of Weak Electron–Phonon Coupling in Nanostructured ZnO Thin Films

**DOI:** 10.3390/nano8080632

**Published:** 2018-08-20

**Authors:** Ashish C. Gandhi, Wei-Shan Yeoh, Ming-An Wu, Ching-Hao Liao, Dai-Yao Chiu, Wei-Li Yeh, Yue-Lin Huang

**Affiliations:** Department of Physics, National Dong Hwa University, Hualien 97401, Taiwan; acg.gandhi@gmail.com (A.C.G.); letherlightness@gmail.com (W.-S.Y.); swarem610823@gmail.com (M.-A.W.); 410214209@gms.ndhu.edu.tw (C.-H.L.); ella9125710@gmail.com (D.-Y.C.); willy730611@gmail.com (W.-L.Y.)

**Keywords:** Raman scattering, quantum confinement, electron–phonon coupling, polar semiconductors, zinc oxide

## Abstract

High-quality crystalline nanostructured ZnO thin films were grown on sapphire substrates by reactive sputtering. As-grown and post-annealed films (in air) with various grain sizes (2 to 29 nm) were investigated by scanning electron microscopy, X-ray diffraction, and Raman scattering. The electron–phonon coupling (EPC) strength, deduced from the ratio of the second- to the first-order Raman scattering intensity, diminished by reducing the ZnO grain size, which mainly relates to the Fröhlich interactions. Our finding suggests that in the spatially quantum-confined system the low polar nature leads to weak EPC. The outcome of this study is important for the development of nanoscale high-performance optoelectronic devices.

## 1. Introduction

Bulk ZnO is an n-type semiconductor material with a direct wide band gap energy (3.37 eV) and large exciton binding energy (60 meV) at room temperature. These properties make ZnO one of the most promising oxide semiconductors for high-performance optoelectronic devices, such as touch screens, liquid crystal displays, light-emitting diodes, chemical/biological sensors, dye-sensitized solar cells, and piezoelectric devices [1,2,3]. The optical properties of nanoscale devices can be controlled by tailoring the average size, shape, and surface modifications of nanometer-sized ZnO crystals [4,5,6]. However, at the nanoscale, the optical and the electrical properties, such as the energy relaxation rate of excited carriers and phonon reproduction of excitons in the photoluminescence (PL), as well as Raman scattering, are greatly influenced by the electron–phonon coupling (EPC) in semiconductor materials [7]. For instance, the electric field within a material correlates to Coulomb interactions with the exciton, and the strength of EPC will be enhanced if the wavelength of the phonon vibration is comparable to the spatial extent of the exciton [8,9,10,11,12,13]. Such a quantum-confined spatial system of semiconductors is possible to prepare. However, the optical and the electrical properties of such system differ significantly from their bulk counterparts.

In the past, both theoretical and experimental studies have been done to estimate the EPC strength and its dependence on the crystallite size of semiconductor nanocrystals [7,8,9,10,11,12,13,14,15,16]. Theoretically, it has been suggested that the electron–longitudinal optical (LO) phonon coupling should vanish with decreasing nanocrystal size by using a simple charge neutrality model [9]. However, using the non-parabolicity of the bands, it has been shown that EPC strength increases when decreasing the material size [10,11]. Experimentally, using Raman spectroscopy, it has been observed that Fröhlich interactions play an important role in defining the strength of EPC in various ZnO nanocrystals and its value decreases when reducing the crystallite size. However, the dependence of EPC strength on the nanocrystal shape and size of wide bandgap ZnO is still not completely understood [7,17,18,19,20,21,22,23]. Therefore, deliberate control of size and shape of ZnO nanostructures is of particular interest for the understanding of the fundamental physics of ZnO nanocrystals and for their application in functional devices.

The sputtering method has many advantages including better film growth control, uniformity, repeatability, low-temperature deposition, and large-scale stability. The properties of sputtered ZnO thin films not only depend on the deposition parameters (RF power, pressure, substrate temperature, ambient atmosphere), but also on the post-deposition processes such as thermal treatment [24,25,26]. However, the effect of post-annealing on the ZnO film properties has not often been reported in the literature. In this study, we report on the effect of post-annealing on the structural and the optical properties of the magnetron sputtering deposited ZnO thin films. The main focus of this study is to examine the influence of the grain size on the evolution of phonon confinement and the strength of EPC using Raman spectroscopy. Raman spectroscopy is a versatile and fast nondestructive characterization technique sensitive to distortions in the crystal lattice, crystal defects, and phase transformation [27]. Based on inelastic light scattering, it can provide information about the phonon vibrational and rotational mode properties of post-annealed ZnO to get insight into the effect of size reduction on the EPC in ZnO nanocrystals. The results of crystalline structure, grain size, surface morphology, defects, and optical properties of both as-deposited and post-annealed ZnO thin films have been investigated.

## 2. Materials and Methods

The vapor-phase deposition was performed with reactive sputtering on sapphire substrates with Al_2_O_3_ (0001) normal orientation using a Zn target of purity 99.995% located about 8 cm away from the substrates and a mixture gas with argon:oxygen = 4:1. During the deposition, a total pressure was maintained at 50 mTorr and monitored by an absolute capacitance manometer. A direct-current power supply was used to drive the magnetron sputtering plasma at 80 W. The substrate was rotated at a speed of three turns per minute to eliminate the effects of slanted deposition. A calibrated quartz crystal microbalance (Model TM-350, Matek Inc., Cypress, CA, USA) operating at 6 MHz was taken to follow the deposited mass in real time, realizing a comparable thickness of 300 nm for each film. The sputtered films (ZnO/ALO) were annealed in air for one hour at temperatures ($T_{A}$) from 100 to 800 °C in a step of 100 °C followed by characterizations of structure and properties at room temperature. Structural and surface morphological analysis of films was carried out using X-ray diffraction (XRD, Rigaku D/Max-2500V X-ray Diffractometer, The Woodlands, TX, USA) with CuK *α* radiation (wavelength: 1.54178 Å) and scanning electron microscopy (SEM) taking 15 keV incidence beam for secondary-electron imaging on JEOL JSM-7000F (Peabody, MA, USA) equipped with a field emission electron source. The mean diameter distribution was estimated from relative SEM images using Nano Measurer software (Nano measurer 1.2.5, Jie Xu, Fudan University, Shanghai, China). Raman scattering was utilized to study the defects and the effect of reduced grain size on phonon confinement and the strength of EPC. Scattering spectra were recorded with a Jobin Yvon T64000 spectrometer (Horiba Scientific, Paris, France) using a continuous-wave laser of 325 nm. The excitation laser power on the sample was ~4 mW with 1 μm diameter of the laser beam spot. The full profile fitting of Raman spectra was carried out using Fityk 0.9.3 software [28].

## 3. Results and Discussion

### 3.1. Morphology and Structural Characterization

**Figure 1:**
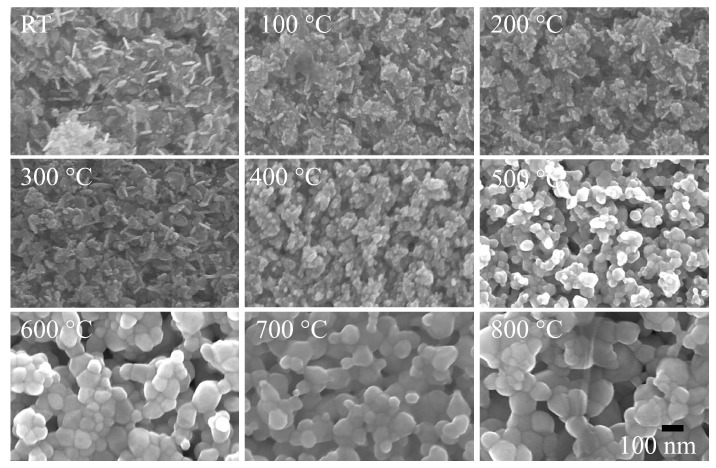
Scanning electron microscopy (SEM) images of RT and 100 °C to 800 °C annealed ZnO/ALO films. (All the images are taken at the same magnification (×10^5^) with a scale bar of 100 nm shown in the image of 800 °C).

Figure 1 shows SEM images of as-deposited room temperature (RT) and 100 to 800 °C annealed ZnO/ALO films (in a step of 100 °C), revealing granular nature within nanometric range and an increase of grain size with the increase of T_A_. Uniformly distributed nanoplate-like grains of ~10 to 15 nm thickness and ~50 to 80 nm length have been seen from RT and 100 to 300 °C annealed films. Annealing at temperatures from 400 to 500 °C resulted in the transformation of nanoplates into pseudospherical nanoparticles, forming agglomerated sphere-like nanoparticles. However, the annealing at and above 600 °C resulted in the formation of films with interconnected granular surface morphology. The mean diameter <*d*> of asymmetrically distributed nanoparticles was obtained by fitting the log-normal distribution function $f\left(d\right)=\frac{1}{\sqrt{2\pi }d\sigma }\exp\left[-\frac{\left(\ln d-\ln\langle d\rangle \right)}{2\sigma^{2}}\right]$ to the histogram obtained from SEM images, where *σ* is the standard deviation of the fitted function shown in Figure S1. The fitted values of (〈d〉, σ) vary from ~(13 nm, 0.16) to (65 nm, 0.42) with the increase of *T*_A_ from RT to 800 °C (see Table S1). The value of 〈d〉 ~13 nm, which is the width of the nanoplates, remains independent of *T*_A_ up to 300 °C; above that a drastic increase in the grain size can be seen, reaching a saturation value of ~62 nm at *T*_A_ 600 °C. From the fitted values of σ, it appears that the distribution of small-sized particles is confined within a narrow range at low *T*_A_ and evolves with an increase of *T*_A_.

**Figure 2:**
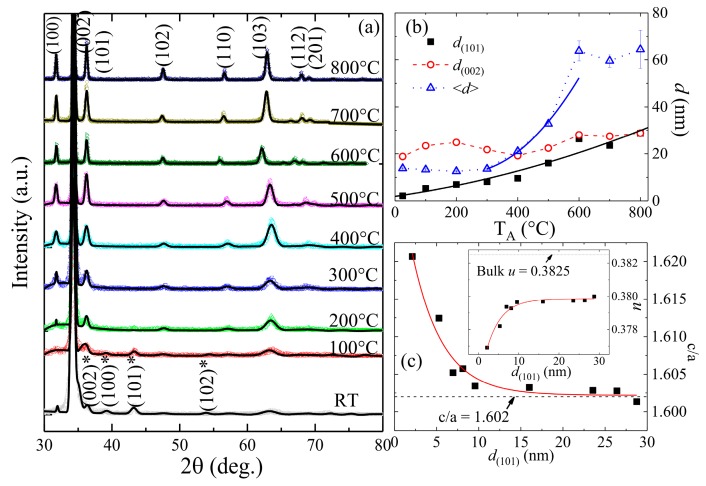
(**a**) “F(calc) weighted” LaBail extraction analysis [32] (solid line) for the X-ray diffraction (XRD) pattern (crosses) of the RT, and 100 to 800 °C annealed ZnO/ALO films (bottom to top) with Bragg reflections of ZnO and Zn (star-marked) phases indicated; (**b**) T_A_-dependency of *d*_(002)_, *d*_(101)_ calculated from XRD patterns and the fitted values of <*d*> obtained from SEM images. Black colored solid lines represent the growth law fitting to *d*_(101)_; (**c**) Grain-sized *d*_(101)_ dependence of the ratio of lattice constants *c*/*a*, where the horizontal lines represent the value of bulk ZnO, and the solid line denotes the exponential fit. The inset in (**c**) shows the corresponding evolution of the internal parameter *u*.

Figure 2a shows XRD spectra for RT and 100 to 800 °C annealed ZnO/ALO films, taken under a fixed glancing incident angle of 3°. All Bragg reflections from RT film were identified to emerge from wurtzite ZnO (JCPDS 036-1451), and simple hexagonal Zn (JCPDS PDF#00-004-0831) [29], indicating the high purity of the vapor-phase deposited film. The annealing at temperatures above 100 °C resulted in complete oxidation of Zn and the formation of pure ZnO films. This was also supported by energy-dispersive X-ray spectroscopy, showing that only Zn, O, Al, and C are detectable in the films (data not shown), where Al and C came from the sapphire substrates and surface adsorbents respectively. The crystallinity of film improves with an increase of T_A_, resulting in an enhanced Bragg maximum and reduced spectral width. The appearance of weak diffraction peaks corresponding to the (100), (101), (102), (110), (103), (112) and (201) planes of ZnO suggested the presence of some randomly oriented grains. However, there was a preferential growth along the (002)_ZnO_ plane’s normal, resulting in the very strong (002) reflection in Figure 2a. The intensity profile of the most intense (002) and (101) reflections can be fitted with a Lorentzian distribution function to determine its full-width at half maximum (FWHM) β. Using Scherrer’s formula: d=kλ/β cosθ (where *k* = 0.94 is a Scherrer’s constant and θ is the Bragg angle), the calculated grain size (*d*_(002)_, *d*_(101)_) of RT, and 100 to 800 °C ZnO films is (2 nm, 19 nm), (5 nm, 23 nm), (7 nm, 25 nm), (8 nm, 22 nm), (10 nm, 20 nm), (16 nm, 22 nm), (26 nm, 28 nm), (24 nm, 27 nm) and (29 nm, 29 nm), respectively (see Table S1). Figure 2b compares the calculated values of *d*_(002)_, *d*_(101)_ and estimated <*d*> from SEM images with respect to *T*_A_. The value of *d*_(002)_ shows a minimum of 20 nm at *T*_A_ = 400 °C, whereas *d*_(101)_ increases with *T*_A_ and at and above 600 °C, *d*_(101)_ ≈ *d*_(002)_. Above finding indicates a change in grain dimensions with aspect ratio *d*_(002)_/*d*_(101)_ decreasing from ~9 to ~1 due to increasing *T*_A_ up to 800 °C. Furthermore, particle size <*d*> ~13 nm obtained from SEM images (RT to 300 °C) is smaller than that of *d*_(002)_ ~20 nm, indicating that nanoplate-like grains exhibit fast grown rate along (002) direction, which is consistent with XRD spectra. This trend is consistent with SEM observations, supporting the idea that ZnO grains have developed from small plate-like grains (which could be oriented along the *c*-axis) to large sphere-like ones above 400 °C. Our findings revealed that much finer nanocrystals can be formed during the reactive deposition of ZnO films as compared with ZnO grains (*d*_(101)_ ~13 to 24 nm) formed through annealing a pure Zn film on Al_2_O_3_(001) substrates (Zn/ALO) with a comparable film thickness for one hour in air at similar temperature *T*_A_ varying from RT to 800 °C. As compared with the ZnO/ALO film, different structural evolution of ZnO from post-annealing of the Zn/ALO film is reflected in the distinct XRD 2-θ scan patterns (Figure S2) as well as the calculated lattice constants and the internal parameter *u* (see the comparison in Table S1 and Figure S3). This difference is thought to be related with the highly anisotropic early-stage growth of ZnO crystallites emerged during reactive sputtering of the ZnO/ALO film leading to an aspect ratio of *d*_(002)_/*d*_(101)_ as high as ~9. Whereas, ZnO crystallites evolved isotropically from nearly-sphere-shaped crystallites with aspect ratios between 0.9 and 1.1 formed during post-annealing of the Zn/ALO film in the air. In fact, ZnO grain sizes in as-deposited films have been observed to depend on sputtering parameters [30,31]. Our observation of average grain sizes down to ~5 nm in low-temperature annealed ZnO/ALO films are consistent with a previous report that fine crystallites are formed during reactive sputtering of ZnO films on Si(100) substrates using oxygen partial pressures below 30% (20% in our case) [30]. The fact that the calculated grain size using XRD is much smaller than the particle size estimated from SEM images is attributed to the formation of multi-grain ZnO particles due to annealing, particularly at temperatures above 400 °C.

For detailed structural investigation of the ZnO/ALO films, “F(calc) weighted” LaBail extraction analysis of all samples was carried out by refining XRD spectra using the GSAS software package, as shown in Figure 2a [32]. We confirmed that the Zn and ZnO phase (space group P6_3_/mmc and P6_3_mc) existed in RT and 100 °C films, whereas there is a single ZnO phase in 200 to 800 °C films. The fitted values of lattice constants (*a* = *b*, *c*) of the ZnO phase obtained from RT, and 100 to 800 °C films, are summarized in Table 1, showing a lattice contraction along the *a = b* axis from RT and 100 °C films, whereas the value obtained from 200 to 800 °C films shows a value very near the bulk value of 3.250 Å. On the contrary, the lattice constant along the *c*-axis shows exponentially decreasing behavior with the increase of *T*_A_ and approaches the bulk value of 5.207 Å around ~500 °C, above which it is almost *T*_A_-independent (see Figure S3a,c). The axial ratio *c*/*a* and the internal parameter *u* (*u* represents the relative position of two hexagonal close-packed sublattices of the wurtzite) can be used as indicators of the polarity as the deviation occurs in the lattice of the wurtzite ZnO structure. Figure 2c shows the $d_{\left(101\right)}$ dependence of fitted values of *c*/*a*, revealing the development of short- to long-range polar interaction with the increase of grain size. The solid curve in Figure 2c denotes an exponential decay function, namely $c/a={\left(c/a\right)}_{\mathrm{Bulk}}+0.034\left(5\right)\exp\left(-d_{\left(101\right)}/\xi_{o}\right)$, where ${\left(c/a\right)}_{\mathrm{Bulk}}=1.602$ represents the ratio of long-range polar interaction and $\xi_{o}=3.6\left(6\right)$ nm is the domain size. The obtained domain size is very near that of the quantum-confined dots (~3.5 nm), from which a very weak EPC strength of ~0.39 has been reported [18]. Ignoring the slight length difference between the Zn–O bond along c-axis and the others, the internal parameter can be estimated by $u=\frac{1}{3}\left(\frac{a^{2}}{c^{2}}\right)+\frac{1}{4}$. Taking the fitted values of lattice parameters *a* and *c*, changes in the estimated *u* with respect to *d*_(101)_ (shown in the inset of Figure 2c) are found to evolve from 0.3769 (RT) and show an exponentially increasing behavior with size, resulting in a saturation value of ~0.3800 at ~10 nm (*T*_A_ = 400 °C), which is smaller than the reported bulk value of 0.3825. In contrast, as discussed before, the annealing of pure Zn films (Zn/ALO) at 100–400 °C for 1 h in air has led to the formation of much larger grains of ZnO (*d*_(100)_ ≈ 11–14 nm) with aspect ratios of ~0.9–1.1 and significantly lower values of the internal parameter *u* ~0.3797–0.3798 (see Table S1 and Figure S3d). Therefore, both the surface dipole-induced electrostatic effects of the polar wurtzite structure and the vapor-phase ambient conditions during the magnetron sputtering were thought to dominate the anisotropic growth of ZnO nanocrystals during deposition. The observed increase of *c*/*a* and the very low value of *u* in fine-sized granular films (<10 nm) could be due to the effect of compressive strain. The strain (ε) and stress (σ) in the films, along with the *c*-axis, can be estimated using $\varepsilon =\left(c_{\mathrm{film}}-c_{\mathrm{bulk}}\right)/c_{\mathrm{bulk}}$ and $\sigma =-2.33\times 10^{11}\mathrm{GPa}\cdot \left(c_{\mathrm{film}}-c_{\mathrm{bulk}}\right)/c_{\mathrm{bulk}}$, where $c_{\mathrm{film}}$ and $c_{\mathrm{bulk}}=5.207\;Å$ are the lattice parameters of the film and unstrained ZnO, respectively. The calculated values of ε, σ summarized in Table 1 show decreasing behavior with the increase of grain size. Overall trends seen from SEM images, grain size, and lattice constant can be understood by considering that increasing *T*_A_ at and above 400 °C resulted in morphology transition accompanied by lattice relaxation towards equilibrium bulk state as indicated with the bulk value of *c*/*a* or *u*. The above structural finding indicates that the effect of quantum confinement in RT and low-temperature annealed films could also influence the band gap shift and EPC strength.

### 3.2. Electron–Phonon Coupling in ZnO Films

**Figure 3:**
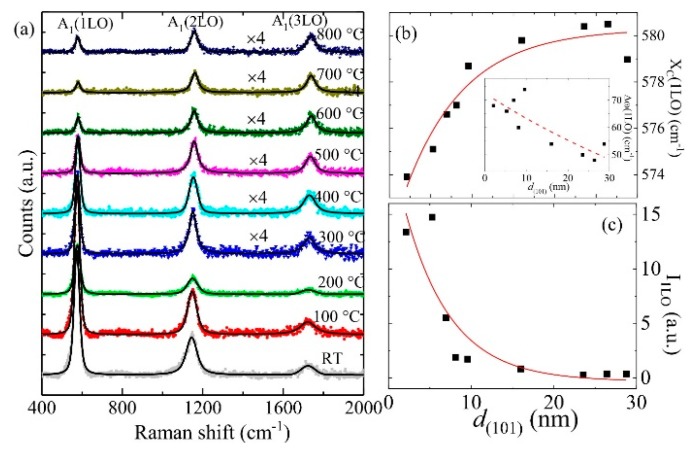
(**a**) Raman spectra of RT and 100 to 800 °C annealed ZnO/ALO films, where the solid line represents the fit using Voigt function; (**b**) The *d*_(101)_ dependency of peak center of A_1_(1LO) phonon mode where the solid line represents the fit. Inset of the figure shows *d*_(101)_ dependency of the spectral width Δω(1LO), where the dashed line is a guide for the eye; (**c**) The *d*_(101)_ dependency of the Raman scattering intensity I_1LO_, where the solid line is a guide for the eye.

An intense multiphonon scattering of the RT and 100 to 800 °C annealed ZnO/ALO films with various grain sizes was observed with the Raman spectra in Figure 3a, where the three major bands were attributed to longitudinal optical A_1_(LO) polar symmetry modes and its overtones A_1_(2LO, 3LO). It is interesting to note that as *T*_A_ increased, the intensities of the A_1_(LO) mode and their overtones decreased, with the spectral width narrowing down. The anomalous behavior was analyzed quantitatively using a profile fitting method. The solid line in Figure 3a represents the fit using the Voigt function covering the whole spectrum, and the fitting parameters are summarized in Table S2. Based on the reported zone-center optical phonon frequencies in ZnO, Raman spectra obtained from RT ZnO film exhibited prominent maxima at 573.9(1) cm^−1^, 1144.7(5) cm^−1^ and 1724(2) cm^−1^ identified with A_1_(1LO), A_1_(2LO), and A_1_(3LO) respectively, which are consistent with that observed on sol-gel derived ZnO quantum dots of size down to ~3.5 nm [18]. Figure 3b depicts the *d*_(101)_ dependency of the peak center of A_1_(1LO), which can be described very well using $X_{C}\left(1\mathrm{LO}\right)=X_{C0}\left(1\mathrm{LO}\right)\left[1-0.016\;\exp\left(-\left(d_{\left(101\right)}/d_{C}\right)\right)\right]$, where $X_{C0}\left(1\mathrm{LO}\right)$ = 580 cm^−1^ is the bulk value of ZnO and $d_{C}=7\left(2\right)$ nm. Compared with that of the ZnO bulk, a redshift ranging from ~0 to 6 cm^−1^ was obtained as the grain size decreased from 29 to 2 nm. Inset of Figure 3b shows the obtained increasing behavior of full-width at half-maximum (Δω) of A_1_(1LO) mode with the decrease of grain size. A similar redshift and the broadening due to decreasing grain size was also observed from the overtones of A_1_(1LO) mode. Figure 3c shows the *d*_(101)_ dependency of the integrated intensity of A_1_(LO) mode (I_1LO_), where the solid line is a guide for the eye. An exponential decay in the intensity can be seen with the increase of grain size (i.e., increase of *T*_A_). Such a pronounced spectral redshift, broadening, and the asymmetry of A_1_ mode and its overtones from small sized grains could result either from phonon localization due to intrinsic defects or the spatial confinement within the grain boundaries. However, from the PL spectra of the ZnO/ALO films, we have observed (i) a blueshift in UV emission from 3.25 eV to 3.30 eV and (ii) a redshift in discernible broad and intense green band emission (1.8–2.7 eV) with the decreasing grain size from 10 to 2 nm (data not shown). Since, exciton Bohr radius of bulk ZnO is 2.34 nm (i.e., diameter 4.68 nm), the carrier confinement in the ZnO films with grain size below 9(1) nm is in the moderate to strong confinement regimes. Therefore, the observed blueshift of UV emission with decreasing grain size can be ascribed to quantum confinement effect, whereas redshift of green band emission to the high density of oxygen vacancies at the surface of the grains. Interestingly, both UV and green band emission do not show any size dependency above 10 nm (further details about PL of granular ZnO films will be published in the near future). The above finding suggests that the *n*-phonon process is contributed to the Raman scattering cross section of low annealed films, which occurs when the energy of incoming or scattered photon matches the real electronic states in the quantum-confined material.

**Figure 4:**
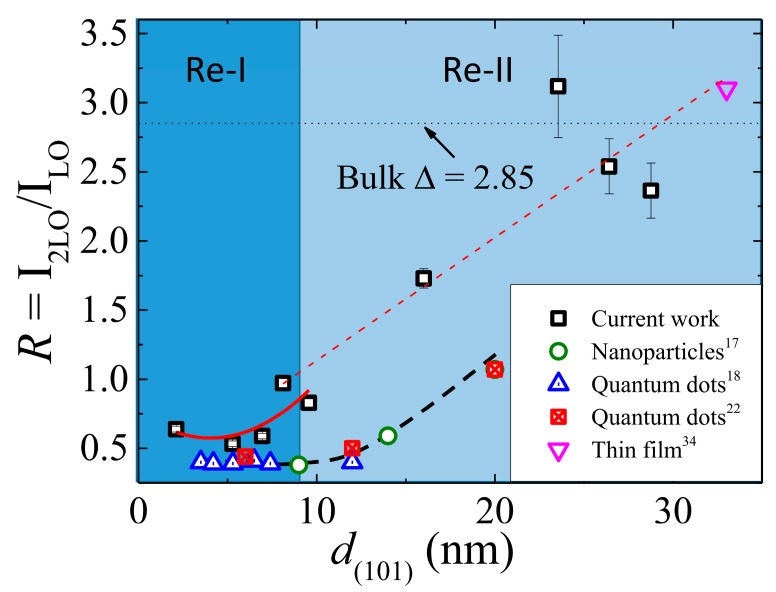
A plot of *d*_(101)_ dependency of EPC strength in the current work compared with that reported for nanoparticles [17], quantum dots [18,22], and thin granular film [34], where the horizontal dotted line indicates the bulk ZnO value of 2.85. The solid red color line represents a fit as discussed in the text, whereas the straight red and black dashed lines are guides for the eye.

The ratio of the relative cross-sections of the first- and second-order Raman scattering A_1_(2LO) and A_1_(1LO) modes, *R* = I_2LO_/I_1LO_, can be used to estimate the EPC strength, which is related to the Huang–Rhys parameter *S* by taking the scattered intensity $I_{n\mathrm{LO}}\propto S^{n}e^{-S}/n!$ within the Franck–Condon approximation [33]. Along with the grain size dependency of EPC strength observed in this work, Figure 4 also depicts the size dependency of reported values of EPC strength of nanoparticles (9 to 20 nm) [17], quantum dots [18,22] (3.5 to 20 nm), and granular thin film (33 nm) [34]. It is interesting to note that quantum dots with size below 12 nm show a lowest, almost a constant value of EPC strength ~0.4 and above which it increases exponentially, whereas a highest value of EPC from the granular thin film approaches 3.1 [18]. Therefore, the EPC strength obtained from granular ZnO films can be split into two regions, namely, Re-I (~2 to 9 nm) and Re-II (~9 to 29 nm). Contrary to size dependency of quantum dots, the grain size dependency of EPC strength in Re-I shows shallow deep around ~5 nm, which can be described as $R=0.76-0.09d_{\left(101\right)}+0.01d_{\left(101\right)}^{2}$ (represented by the solid curve in Figure 4). However, in Re-II, above ~9 nm, a drastic increase in the EPC strength with value approaching towards a bulk value of 2.85 (represented by a horizontal dotted line) can be seen, where dashed line is guided for the eye. The deformation potential and the Fröhlich potential are responsible for the size dependency of the EPC strength. The TO phonon mode includes a contribution from the deformation potential, involving short-range interaction between electrons and the lattice displacements, whereas the LO phonon mode includes contributions from both the potentials that involve the long-range interaction generated by the macroscopic electric field associated with the LO phonons. However, in the present set of ZnO films, under resonant conditions, the intensity of the LO phonon greatly increases with the decrease of grain size, and that of the TO phonon is almost insensitive. Therefore, the low value of EPC obtained from the spatially quantum-confined grains in Re-I can be attributed to less polar nature as observed from XRD analysis, whereas the linear increasing behavior of EPC in Re-II signaling strong size dependency is mainly related to the long-range Fröhlich interactions.

Structural reconstruction has been proposed to take place while annealing reactively sputtered ZnO films with T_A_ exceeding the melting point of zinc (420 °C for the bulk) due to forming films in a melted state, whereas higher T_A_ is needed for recrystallization [35]. In contrast to a recrystallization process, we propose that a structural reconstruction process takes place accompanied with out-diffusion of native defects thermally activated at high T_A_. This view was corroborated with correlated observations with *T*_A_ ~ 600 °C: (i) annealing at and above 600 °C resulted in the formation of films with well interconnected granular (~62 nm) surface morphology (ii) retrogressive increase of oxygen vacancy (V_O_) density in the surface region revealed by XPS (data not shown), and (iii) drastic enhancement of the EPC strength revealed by Raman scattering. Since no surface vibration modes were detected in our study, Raman scattering events were thought to have taken place mainly inside the bulk. The proposed thermally activated out-diffusion is consistent with recently reported density functional theory calculations supporting an energetically favorable migration of V_O_ in the direction from bulk to surface in bare ZnO nanowires [36]. Our observations were self-consistent in that bulk EPC property was approached at high T_A_ when lattice relaxation revealed with XRD also proceeded in parallel towards bulk state as discussed in Figure 2c. It is noted, however, that a direct comparison of EPC strengths derived for nanostructures with that of bulk is inappropriate and could lead to conceptual confusion [7,37].

## 4. Conclusions

Granular ZnO film with nanoplate-like morphology and strong preferential growth along the (002) plane’s normal was grown by reactive sputtering technique. Annealing from 100 to 800 °C in an ambient atmosphere leads to an increase of crystalline size *d*_(101)_ from 2 to 29 nm and a morphology transformation of nanoplate-like shapes to well inter-connected sphere-like grains. The structural analysis carried out by “F(calc) weighted” LaBail extraction analysis of XRD spectra reveal low polar nature of small-sized granular films. The EPC strength deduced from the ratio of the second- to the first-order Raman scattering intensity shows a linear decreasing behavior with the decrease of grain size down to 10 nm, in principle as a result of the Fröhlich interaction. However, below a critical size of 9 (1) nm, a shallow valley with a minimum of EPC strength ~0.53 was obtained around 5 nm. Our finding suggests that in the spatially quantum-confined system the low polar nature leads to weak EPC strength. The outcome of this study is important for the future development of nanoscale high-performance optoelectronic devices.

## Figures and Tables

**Table 1:** Lattice constants *a* = *b* and *c* for Zn and ZnO phases and the internal parameter *u*, strain ε , stress 𝜎, and parameters wRp and Rp for fitting the XRD patterns of the ZnO/ALO films based on the “F(calc) weighted” LaBail extraction analysis [32].

*T*_A_ (°C)	Zn		ZnO					wRp	Rp	χ^2^
	*a* = *b* (Å)	*c* (Å)	*a* = *b* (Å)	*c* (Å)	u	ε (%)	σ (GPa)			
25 (RT)	2.6531(11)	5.1315(16)	3.2358(11)	5.2442(3)	0.3769	0.71	−1.66	0.2638	0.1538	6.132
100	2.6569(13)	5.0304(24)	3.2462(8)	5.2343(7)	0.3782	0.52	−1.22	0.3403	0.2317	5.741
200			3.2514(8)	5.2191(2)	0.3794	0.23	−0.54	0.2639	0.1794	4.989
300			3.2495(7)	5.2179(2)	0.3793	0.21	−0.49	0.2492	0.1597	5.448
400			3.2525(6)	5.2151(5)	0.3797	0.16	−0.36	0.3169	0.2233	4.235
500			3.2449(7)	5.2023(12)	0.3797	−0.09	0.21	0.3092	0.204	3.878
600			3.2485(5)	5.2066(9)	0.3798	−0.01	0.02	0.3743	0.2436	3.426
700			3.2509(2)	5.2106(2)	0.3797	0.07	−0.16	0.3084	0.1907	4.043
800			3.2488(4)	5.2024(6)	0.3800	−0.09	0.21	0.3515	0.2318	3.3

## Supplementary Materials

The following are available online at http://www.mdpi.com/2079-4991/8/8/632/s1, Figure S1: Size-distribution histograms with log-normal fitting for the RT and 100 °C to 800 °C (T_A_) annealed ZnO/ALO films; Figure S2: Rietveld analyses (solid line) of XRD pattern (crosses) for the 100 °C to 800 °C (T_A_) annealed Zn/ALO films; Figure S3: Lattice parameters (a) *a* = *b*, (b) *c*, their ratio (c) *c*/*a*, and the internal parameter (d) *u* of the ZnO/ALO and Zn/ALO films in dependence of T_A_ (a, b) and *d*_(101)_ (c, d) respectively; Table S1: 〈d〉, σ obtained from the log-normal fitting in Figure S1 for the ZnO/ALO films, *d*_(002)_ and *d*_(101)_ calculated using the Scherrer’s formula for the ZnO/ALO and Zn/ALO films; Table S2: Fitted values of Raman mode frequencies X, spectral widths Δω, and the intensity ratio *R* = I_2LO_/I_1LO_ for the ZnO/ALO films.

## References

[1] Muchuweni E., Sathiaraj T.S., Nyakotyo H. (2016). Effect of gallium doping on the structural, optical and electrical properties of zinc oxide thin films prepared by spray pyrolysis. Ceram. Int..

[2] Muchuweni E., Sathiaraj T.S., Nyakotyo H. (2016). Low temperature synthesis of radio frequency magnetron sputtered gallium and aluminium co-doped zinc oxide thin films for transparent electrode fabrication. Appl. Surf. Sci..

[3] Lee J.-H., Ko K.-H., Park B.-O. (2003). Electrical and optical properties of ZnO transparent conducting films by the sol–gel method. J. Cryst. Growth.

[4] Yang Y., Yan H., Fu Z., Yang B., Zuo J. (2006). Correlation between 577 cm^−1^ Raman scattering and green emission in ZnO ordered nanostructures. Appl. Phys. Lett..

[5] Chassaing P.-M., Demangeot F., Paillard V., Zwick A., Combe N., Pagès C., Kahn M.L., Maisonnat A., Chaudret B. (2007). Raman study of *E2* and surface phonon in zinc oxide nanoparticles surrounded by organic molecules. Appl. Phys. Lett..

[6] Lu J.G., Ye Z.Z., Zhang Y.Z., Liang Q.L., Fujita S., Wang Z.L. (2006). Self-assembled ZnO quantum dots with tunable optical properties. Appl. Phys. Lett..

[7] Wang R.P., Xu G., Jin P. (2004). Size dependence of electron-phonon coupling in ZnO nanowires. Phys. Rev. B.

[8] Alivisatos A.P., Harris T.D., Carroll P.J., Steigerwald M.L., Brus L.E. (1989). Electron–vibration coupling in semiconductor clusters studied by resonance Raman spectroscopy. J. Chem. Phys..

[9] Schmitt-Rink S., Miller D.A.B., Chemla D.S. (1987). Theory of the linear and nonlinear optical properties of semiconductor microcrystallites. Phys. Rev. B.

[10] Marini J.C., Stebe B., Kartheuser E. (1994). Exciton-phonon interaction in CdSe and CuCl polar semiconductor nanospheres. Phys. Rev. B.

[11] Nomura S., Kobayashi T. (1992). Exciton—LO-phonon couplings in spherical semiconductor microcrystallites. Phys. Rev. B.

[12] Shiang J.J., Risbud S.H., Alivisatos A.P. (1993). Resonance Raman studies of the ground and lowest electronic excited state in CdS nanocrystals. J. Chem. Phys..

[13] Shiang J.J., Wolters R.H., Heath J.R. (1997). Theory of size-dependent resonance Raman intensities in InP nanocrystals. J. Chem. Phys..

[14] Haiming F., Bingsuo Z., Yulong L., Sishen X. (2006). Size effect on the electron–phonon coupling in CuO nanocrystals. Nanotechnology.

[15] Kelley A.M. (2011). Electron-Phonon Coupling in CdSe Nanocrystals from an Atomistic Phonon Model. ACS Nano.

[16] Murphy-Armando F., Fagas G., Greer J.C. (2010). Deformation Potentials and Electron-Phonon Coupling in Silicon Nanowires. Nano Lett..

[17] Cheng H.-M., Lin K.-F., Hsu H.-C., Lin C.-J., Lin L.-J., Hsieh W.-F. (2005). Enhanced Resonant Raman Scattering and Electron−Phonon Coupling from Self-Assembled Secondary ZnO Nanoparticles. J. Phys. Chem. B.

[18] Cheng H.-M., Lin K.-F., Hsu H.-C., Hsieh W.-F. (2006). Size dependence of photoluminescence and resonant Raman scattering from ZnO quantum dots. Appl. Phys. Lett..

[19] Hsieh W.F., Cheng H.M., Lin K.F., Hsu H.-C. Size dependence of band gap variation and electron-phonon coupling in ZnO Quantum Dots. Proceedings of the Pacific Rim Conference on Lasers and Electro-Optics.

[20] Lin K.-F., Cheng H.-M., Hsu H.-C., Hsieh W.-F. (2006). Band gap engineering and spatial confinement of optical phonon in ZnO quantum dots. Appl. Phys. Lett..

[21] Ojha A.K., Srivastava M., Kumar S., Hassanein R., Singh J., Singh M.K., Materny A. (2014). Influence of crystal size on the electron-phonon coupling in ZnO nanocrystals investigated by Raman spectroscopy. Vib. Spectrosc..

[22] Ray S.C., Low Y., Tsai H.M., Pao C.W., Chiou J.W., Yang S.C., Chien F.Z., Pong W.F., Tsai M.-H., Lin K.F. (2007). Size dependence of the electronic structures and electron-phonon coupling in ZnO quantum dots. Appl. Phys. Lett..

[23] Gaikwad S.S., Gandhi A.C., Pandit S.D., Pant J., Chan T.-S., Cheng C.-L., Ma Y.-R., Wu S.Y. (2014). Oxygen induced strained ZnO nanoparticles: An investigation of Raman scattering and visible photoluminescence. J. Mater. Chem. C.

[24] Ismail A., Abdullah M.J. (2013). The structural and optical properties of ZnO thin films prepared at different RF sputtering power. J. King Saud Univ. Sci..

[25] Husna J., Aliyu M.M., Islam M.A., Chelvanathan P., Hamzah N.R., Hossain M.S., Karim M.R., Amin N. (2012). Influence of Annealing Temperature on the Properties of ZnO Thin Films Grown by Sputtering. Energy Procedia.

[26] Gardeniers J.G.E., Rittersma Z.M., Burger G.J. (1998). Preferred orientation and piezoelectricity in sputtered ZnO films. J. Appl. Phys..

[27] Gandhi A.C., Pant J., Pandit S.D., Dalimbkar S.K., Chan T.-S., Cheng C.-L., Ma Y.-R., Wu S.Y. (2013). Short-Range Magnon Excitation in NiO Nanoparticles. J. Phys. Chem. C.

[28] Wojdyr M. (2010). Fityk: A General-purpose Peak Fitting Program. J. Appl. Cryst..

[29] Lupan O., Chow L., Chai G., Heinrich H. (2008). Fabrication and characterization of Zn–ZnO core–shell microspheres from nanorods. Chem. Phys. Lett..

[30] Ellmer K. (2000). Magnetron sputtering of transparent conductive zinc oxide: Relation between the sputtering parameters and the electronic properties. J. Phys. D Appl. Phys..

[31] Hong R., Qi H., Huang J., He H., Fan Z., Shao J. (2005). Influence of Oxygen Partial Pressure on the Structure and Photoluminescence of Direct Current Reactive Magnetron Sputtering ZnO Thin Films. Thin Solid Films.

[32] Von Dreele R.B., Larson A.C. (2000). General Structure Analysis System (GSAS), Los Alamos National Laboratory Report LAUR 86–748.

[33] Huang K., Rhys A. (1950). Theory of light absorption and non-radiative transitions in F-centres. Proc. R. Soc. Lond. Ser. A Math. Phys. Sci..

[34] Zhang X.T., Liu Y.C., Zhi Z.Z., Zhang J.Y., Lu Y.M., Shen D.Z., Xu W., Zhong G.Z., Fan X.W., Kong X.G. (2001). Resonant Raman scattering and photoluminescence from high-quality nanocrystalline ZnO thin films prepared by thermal oxidation of ZnS thin films. J. Phys. D Appl. Phys..

[35] Hsieh P.T., Chen Y.C., Wang C.M., Tsai Y.Z., Hu C.C. (2006). Structural and photoluminescence characteristics of ZnO films by room temperature sputtering and rapid thermal annealing process. Appl. Phys. A Mater. Sci. Process..

[36] Deng B., da Rosa A.L., Frauenheim T., Xiao J.P., Shi X.Q., Zhang R.Q., Van Hove M.A. (2014). Oxygen vacancy diffusion in bare ZnO nanowires. Nanoscale.

[37] Kelley A.M. (2010). Electron-Phonon Coupling in CdSe Nanocrystals. J. Phys. Chem. Lett..

