# Supplementary material for: New Insights into the Role of Weak Electron–Phonon Coupling in Nanostructured ZnO Thin Films
Source: Nanomaterials (Basel). 2018 Aug 20;8(8):632. doi: 10.3390/nano8080632 (PMC6116313; doi:10.3390/nano8080632)
Supplement: Supplementary file 1 [file nanomaterials-08-00632-s001.pdf]

Supplementary Materials

New sights into the role of weak electron-phonon coupling in nanostructured ZnO thin film

Ashish C. Gandhi, Wei-Shan Yeoh, Ming-An Wu, Ching-Hao Liao, Dai-Yao Chiu, Wei-Li Yeh, and Yue-Lin Huang*

Size-distribution histograms with log-normal fitting for the RT and 100 °C to 800 °C annealed ZnO films

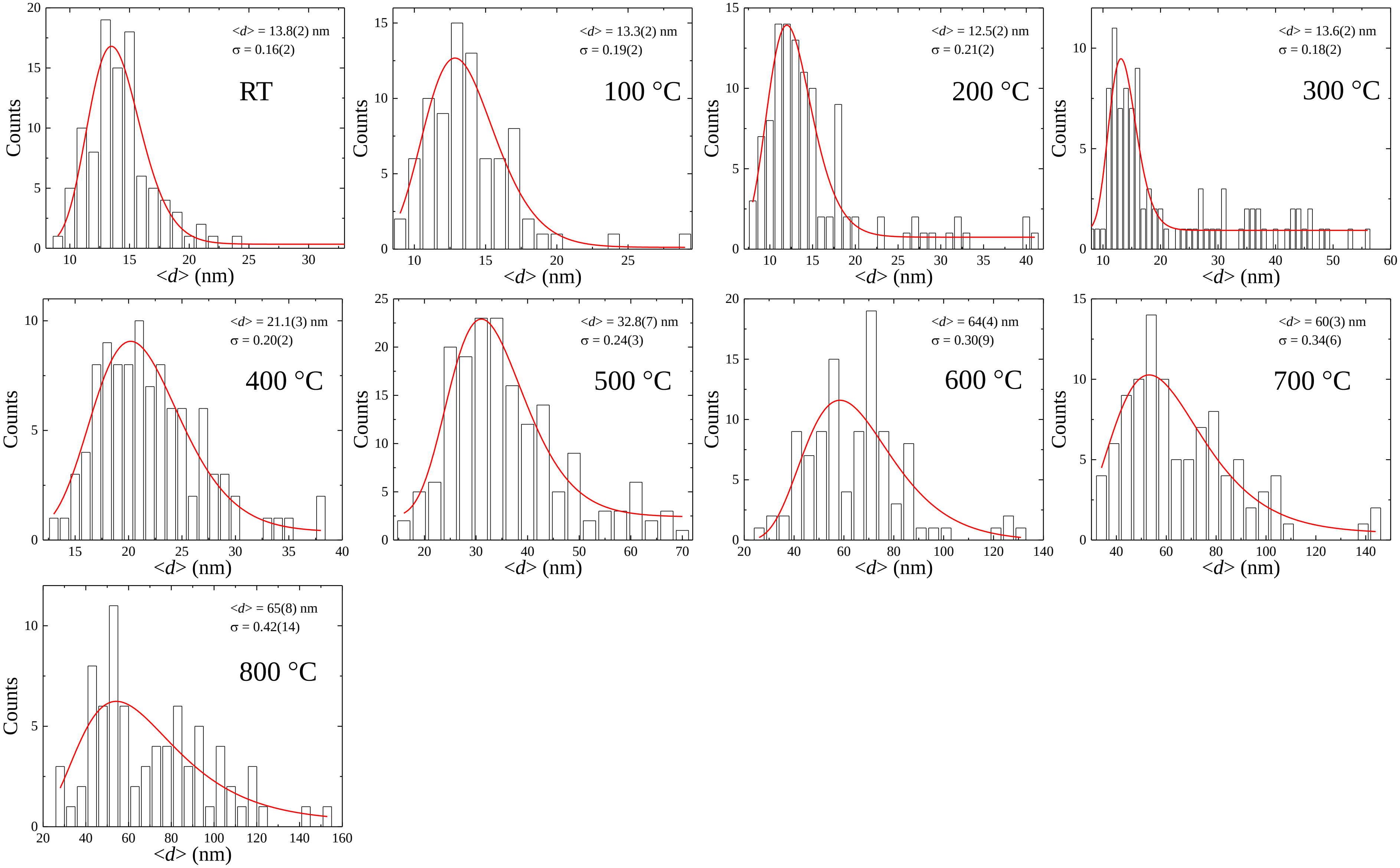

Figure S1 A. C. Gandhi et. al.

Rietveld analyses (solid line) of XRD pattern (crosses) of 100 °C to 800 °C annealed pure Zn films

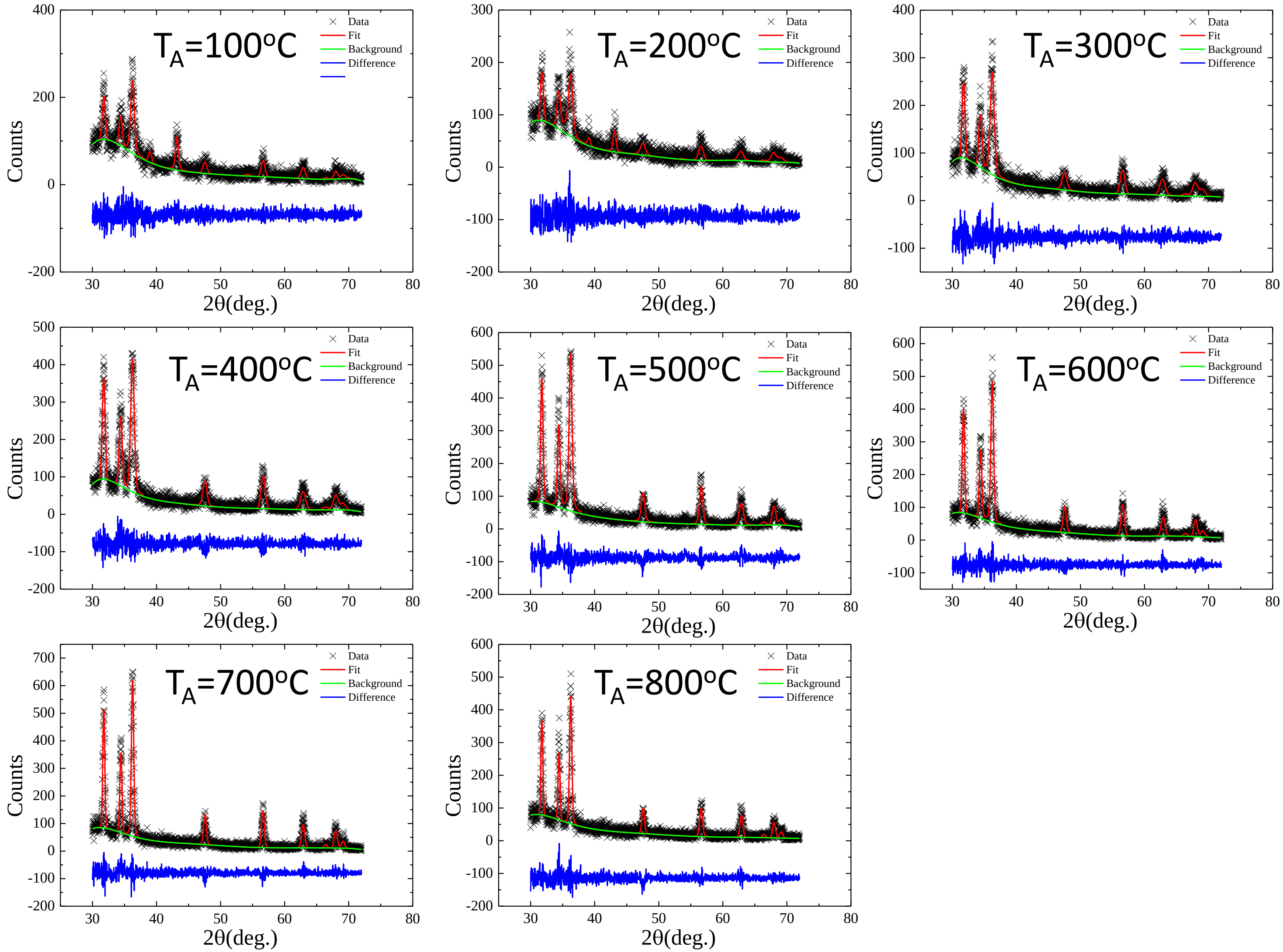

Figure S2 A. C. Gandhi et. al.

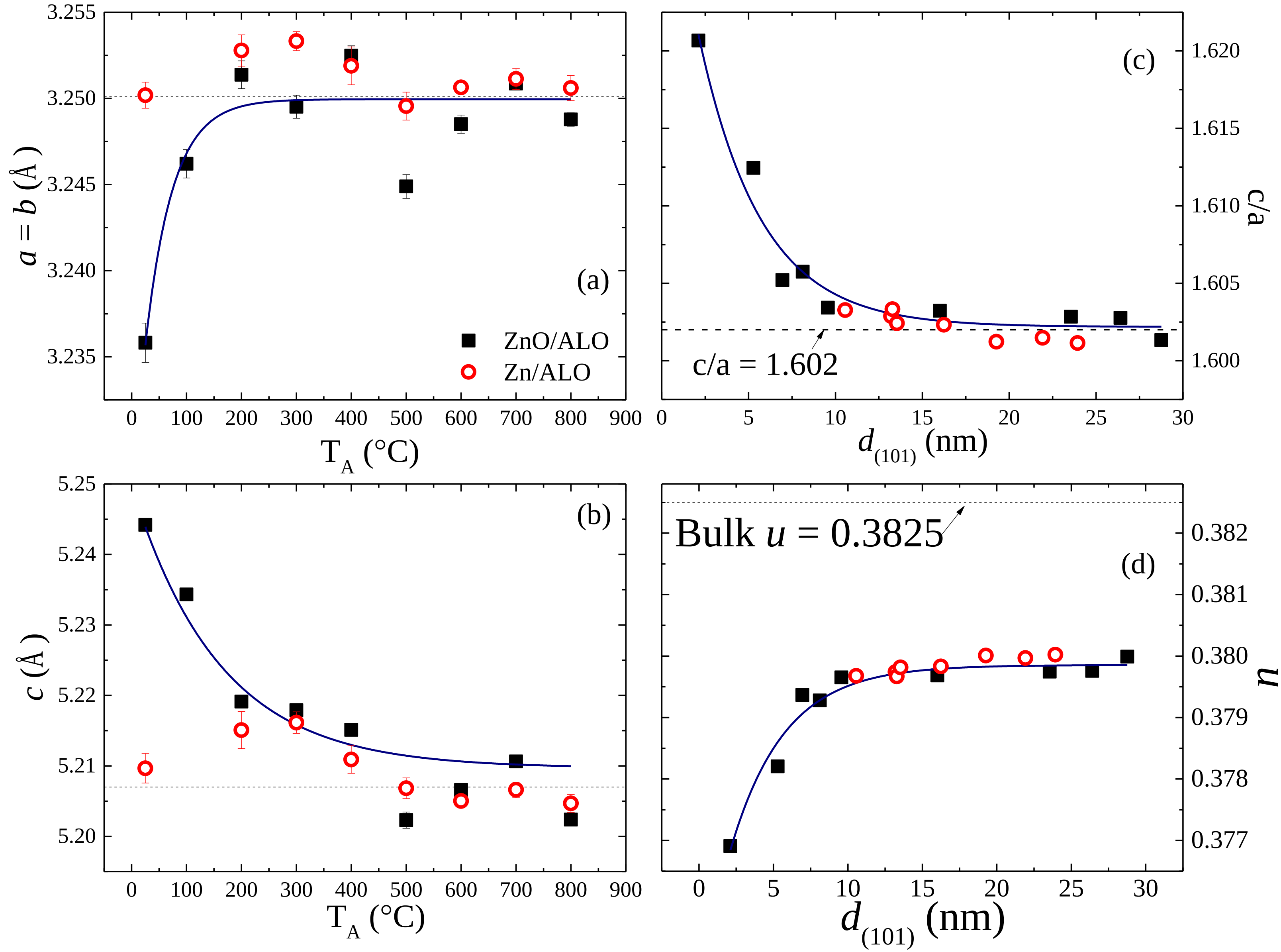

Figure S3 A. C. Gandhi et. al.

		ZnO/ALO			Zn/ALO	
T_A (°C)	<d> (nm)	σ	$d_{(101)}$ (nm)	$d_{(002)}$ (nm)	$d_{(101)}$ (nm)	$d_{(002)}$ (nm)
25 (RT)	13.8(2)	0.16(2)	2.1	18.8	13.2	11.8
100	13.3(2)	0.19(2)	5.3	23.4	….	…..
200	12.5(2)	0.21(2)	6.9	24.9	10.6	11.6
300	13.6(2)	0.18(2)	8.1	21.8	13.3	14.3
400	21.0(3)	0.20(2)	9.6	19.2	13.5	14.9
500	32.8(7)	0.24(3)	16.0	22.4	16.2	19.4
600	64(4)	0.30(9)	26.4	28.1	19.3	19.3
700	60(3)	0.34(6)	23.6	27.5	21.9	23.1
800	65(8)	0.42(14)	28.8	28.8	23.9	26.5

Table S1 A. C. Gandhi et. al.

T_A (°C)	A1(1LO) (cm^{-1})		A1(2LO) (cm^{-1})		A1(3LO) (cm^{-1})		R = $I_{(2LO)}/I_{(1LO)}$
	X_{C1}	Δω(1LO)	X_{C2}	Δω(2LO)	X_{C3}	Δω(3LO)	
25 (RT)	573.9(1)	68	1144.7(5)	132	1724(2)	150	0.64(2)
100	575.1(1)	66	1147.2(5)	110	1722(2)	186	0.53(2)
200	576.6(2)	70	1149.0(6)	126	1725(2)	166	0.59(2)
300	577.0(2)	60	1150.5(5)	88	1729(2)	138	0.97(4)
400	578.7(2)	74	1153.2(4)	100	1730(1)	146	0.83(3)
500	579.8(3)	54	1157.1(4)	92	1735(1)	118	1.73(7)
600	580.5(5)	48	1157.5(5)	86	1740(1)	110	2.54(20)
700	580.4(7)	50	1160.7(5)	84	1739(1)	106	3.12(37)
800	579.0(5)	54	1158.5(6)	84	1739(1)	112	2.36(20)

Table S2 A. C. Gandhi et. al.
